# Effects of hemodialysis with cooled dialysate on high‐sensitivity cardiac troponin I and brain natriuretic peptide

**DOI:** 10.1111/hdi.13039

**Published:** 2022-07-18

**Authors:** Younes Bathish, Karine Beiruti, Hussein Safadi, Adi Sharabi Nov, Elena Bukovetzky, Michael Edelstein, Majdi Halabi, Zeev Israeli

**Affiliations:** ^1^ Nephrology and Hypertension Unit, Cardiology department Ziv Medical Centre Safed Israel; ^2^ Azrieli Faculty of Medicine Bar‐Ilan University Safed Israel; ^3^ Statistical Unit Tel‐Hai Academic College Tel‐Hai Israel

**Keywords:** cardiovascular, hemodialysis delivery systems, outcomes research

## Abstract

**Background:**

Hemodialysis (HD) triggers recurrent and cumulative ischemic insults to the brain and the heart. Cooled dialysate may have a protective effect on major organs and improve hemodynamic tolerability of dialysis. The aim of the study was to compare HD with cooled dialysate with routine dialysis in terms of hemodynamic stability and levels of high‐sensitivity Troponin I (hs‐TnI) and N‐terminal pro b‐type natriuretic peptide (NTproBNP) pre and postdialysis.

**Methods:**

The 45 patients were randomized into two groups. The first group received a 35.5°C dialysate first (hypothermic dialysis) and the second group a 36.5°C dialysate first (routine dialysis). Then groups crossed over, so each group received the alternate dialysate (self‐controls) For each patient, the first sample was collected at the beginning of dialysis, and a second sample was taken at the end of dialysis.

**Results and conclusion:**

hs‐TnI and NTproBNP increased after routine HD by 10.7 ng\ml (*p* < 0.001) and (12.0 pg/μl) (*p* < 0.001), respectively, and by −3.1 ng\ml (*p* = 0.25) and (4.3 pg/μl) (*p* < 0.001), respectively after hypothermic HD. Our study results showed a tendency towards less rise in hsTnI and NTproBNP during hypothermic HD (35.5°C) as compared to routine HD (36.5°C). Neither arm experienced statistically significant changes in blood pressure. Further studies in larger cohorts and long follow up are warranted in order to confirm that lower rise in (hs‐TnI) and NTproBNP actually translate into lower clinical risk for cardiovascular events.

## INTRODUCTION

Hemodialysis (HD) triggers recurrent and cumulative ischemic insults to the brain and the heart.^1^ Cardiovascular disease is the leading cause of morbidity and mortality in patients with chronic kidney disease and end‐stage kidney disease (ESKD) undergoing long‐term HD therapy, accounting for approximately half of all deaths.^2^, ^3^ Most patients experience adverse dialysis‐related symptoms and generally have a poor quality of life.^1^ Myocardial ischemic insult and heart failure, frequently due to left ventricular hypertrophy (LVH), are common in dialysis patients.^4^, ^5^ Repetitive cardiac ischemia during HD leads to cardiac hypertrophy and fibrosis.^1^ Compared with the general population the incidence and prevalence of myocardial injury in dialysis patients is 10–20 times higher because of the frequent association of risk factors for coronary artery disease (CAD) such as diabetes mellitus, hypertension and possibly accelerated atherosclerosis with long‐term HD.^6^ Cardiovascular risk stratification is an important aspect in the management of dialysis patients. It may enable early identification of high‐risk patients, resulting in optimization of therapeutic interventions.^7^

Core body temperature increases during the course of HD because of peripheral vasoconstriction.^8^ Several studies have demonstrated that cooled dialysate (at temperature of 35.5°C) has a protective effect on major organs including the heart and brain. It reduces the incidence of intradialytic hypotension (IDH), improves hemodynamic tolerability of dialysis and reduces HD‐induced recurrent ischemic injury.^8^ IDH is defined by the Kidney Disease Outcomes Quality Initiative (KDOQI) and European Best Practice Guidelines (EBPG) as a decrease in systolic blood pressure ≥20 mmHg or a decrease in mean arterial pressure by 10 mmHg provided that the decrease in blood pressure is associated with clinical events and need for nursing interventions. IDH is a major contributor to increased cardiovascular mortality in ESKD. Dialysate cooling is an inexpensive, easily implementable and well tolerated intervention. Whether dialysate cooling confers long‐term protection to cardiac structure and function remains unknown.^1^

Elevated concentrations of cardiac biomarkers such as troponin T (TnT) and brain natriuretic peptide (BNP) have been reported to be important predictors of cardiovascular events and mortality in dialysis patients.^6^ Troponins are structural proteins involved in the regulation of skeletal and cardiac muscle contractility and cardiac troponins are used as sensitive and specific markers of cardiac injury.^4^ These biomarkers are strongly associated with cardiac hypertrophy and systolic dysfunction and serve as powerful prognostic markers of mortality and adverse cardiovascular outcomes in HD patients.^5^ The majority of patients with ESKD have chronically elevated concentrations of myocardial biomarkers even in the absence of acute coronary syndrome, thus obscuring the diagnosis of myocardial infarction and raising questions concerning the cardiac specificity of these markers.^9^ The causes of elevated concentrations are still unclear but may include injury to skeletal muscle, heart failure, LVH, apoptosis or impaired renal clearance.^5^, ^6^ Conflicting results have been reported regarding the levels of these markers following HD.

The studies that have assessed the impact of cooled dialysate on adverse cardiovascular outcomes are currently limited. Despite evidence suggesting a positive therapeutic effect of cooling dialysate, this procedure is not routinely adopted a standard practice due to inconclusive evidence of the long‐term effects on IDH and the lack of consensus regarding optimal cooling procedures. Further investigation into the use of cooled dialysate should be undertaken to validate this process. The use of specific biomarkers of myocardial infarction to monitor hypothermic HD treatment response has not yet been assessed.

The purpose of this study was to compare HD with cooled dialysate (hypothermic dialysis) and routine HD in terms of changes in myocardial markers hs‐TnI and NTproBNP and blood pressure.

## METHODS

We conducted a crossover, randomized, double‐blind study of end‐stage kidney patients receiving HD in the dialysis unit of Ziv Medical Center, one of the major government hospitals in Northern Israel. The study protocol was approved by the local IRB (0101‐17‐ZIV) and the trial was registered in NIH clinical trials (NCT04929366).

Patients who underwent HD at Ziv dialysis unit for at least 3 months were eligible. The study was performed from October 2018 to December 2018. Exclusion criteria were as follows: (a) patients who had a myocardial infarction in the past 3 months, (b) patients who had chest pain during the week preceding HD, (c) patients in an unstable hemodynamic state and requiring premature dialysis discontinuation, (d) patients who underwent any vascular surgery or intervention in the past month, (e) patients undergoing peritoneal dialysis, (f) critically ill patients, patients unable to consent and patients who are currently pregnant.

Patients were randomized into two arms. Arm 1 was first dialyzed with a 35.5°C dialysate (hypothermic dialysis) and arm 2 with a 36.5°C dialysate (routine dialysis). The arms crossed over, so that at the beginning of the study patients were randomized to either arm and received the alternative regimen in the subsequent dialysis cycle. The dialysis machine was set up by a nurse who were not part of the study. The research team was therefore not aware of which temperature each patient's dialysis was set, and neither was the patient. All patients were dialyzed with heparin as anticoagulant therapy. Dialysis was performed with conventional bicarbonate HD using Fx10 HPS (Fresenius Medical Care AG, Bad Homburg, Germany) synthetic membranes and each HD session were 4 h long. HD was performed according to standard directives including dry weight. No changes to the dialysis protocol except for dialysate temperature were applied during the study. For each patient, four blood pressure measurements were taken; at the beginning of the HD session, after 2 h, after 3 h after 4 h (end of HD). Blood samples were collected during each dialysis sessions. The first sample was taken immediately at the beginning of the dialysis session, and a second sample was taken in the last 5 min of dialysis. Body temperature was measured orally, and blood samples were immediately separated, divided into aliquots and then stored at −70°C until assays were run. Cardiac troponin I concentration (hs‐TnI) was measured using the ARCHITECT STAT (Abbott, USA) highly sensitive assay and BNP with the ARCHITECT BNP (Abbott, USA) assay. Electrocardiograms (ECGs) were performed and electrolytes (K^+^ and Ca^2+^) levels measured on all patients before and in the last 5 min of HD in both arms. ECGs were performed at 10 mm equals to 1 mv at 25 mm/s using Nihon Kohden cardiofaxS device. Intervals of PR, QRS, and QTc were measured. QTc for heart rate was calculated using Bazett's formula.

### Statistical analysis

In terms of analysis, we described categorical variables using frequencies and percentage. For continuous variables, we used median (Me) and range for Table 1 and arithmetic mean and 95% confidence interval for Table 2. The differences (Δ) for each of the variables were calculated as the difference between the two measurement times (after ‐ before). We assessed the distribution of continuous variables using Kolmogorov–Smirnov. Since data were not all normally distributed, we used non parametric tests in our analysis. We determined differences between the two dialysis regimens (35.5 and 36.5°C) in terms of differences in blood pressure measurements and cardiac markers levels using Wilcoxon signed‐rank test (Table 2). Friedman non parametric tests for repeated measures were used in order to assess the three differences of MAP (Table 3). A p‐value of 5% or less was considered statistically significant. The data was analyzed using the SPSS Version 25.

**TABLE 1:** Characteristics of the study patients

Variables	All (*n* = 45)
Age, year (Me, range)	66, 31–83
Gender (*n*, %)	
Male	27, 60.0
Female	18, 40.0
Ethnicity (*n*, %)	
Arab	29, 64.4
Jewish	16, 35.6
HTN (*n*, %)	43, 95.6
Hyperlipidemia, yes (*n*, %)	42, 93.3
Smoking, yes (*n*, %)	13, 28.9
PVD, yes (*n*, %)	8, 17.8
CVD, yes (*n*, %)	20, 44.4
Diabetes, yes (*n*, %)	34, 75.6
CAD, yes (*n*, %)	30, 66.7
History of MI, yes (*n*, %)	15, 33.3
History of PCI, yes (*n*, %)	25, 55.6
CABG, yes (*n*, %)	9, 20.0
Duration of dialysis, years (Me, range)	2.2, 0.3–10.0
Kidney transplant, yes (*n*, %)	2, 4.4
Anti‐aggregates, yes (*n*, %)	35, 77.8
Beta blockers, yes (*n*, %)	32, 71.1
Statins, yes (*n*, %)	28, 62.2
Anti‐coagulants, yes (*n*, %)	6, 13.3
ESKD cause (*n*, %)	
Other	11, 24.4
Diabetes	24, 55.6
Hypertension	4, 8.9
Glomerular Nephritis	5, 11.1
Death, all causes (*n*, %)	16, 35.6

**TABLE 2:** Mean, 95% confidence interval and differences (Δ) in BMI, MAP, temperature of dialysis, hs‐TnI, and NT‐ProBNP, electrolytes and ECG according to type of dialysis and time of measurement

	Routine dialysis (36.5°C)				Hypothermic dialysis (35.5°C)					
Variables	Before	After	Δ	*p* ^a^	Before	After	Δ	*p* ^b^	ΔΔ	*p* ^c^
BMI (kg/h^2^)	28.8 (26.8–30.8)	27.9 (25.9–29.9)	−0.9 (−1.1–[−0.7])	<0.001	28.7 (26.8–30.7)	28.0 (26.0–30.0)	−0.7 (−0.8–[−0.6])	<0.001	−0. 2 (−0.2–0.1)	0.149
Weight (kg)^d^	77.9 (73.0–82.7)	75.6 (70.6–80.6)	−2.4^d^ (−2.9–[−1.9])	<0.001	77.6 (72.7–82.4)	75.6 (70.6–80.5)	−2.0^d^ (−2.2–[−1.7])	<0.001	−0.4 (−0.9–0.1)	0.136
Systolic BP (mm Hg)	138 (132–146)	134 (126–142)	−5 (−13–2.5)	0.311	142 (135–149)	141 (133–149)	−2 (−10–6)	0.615	−2 (−13–10)	0.583
Diastolic BP (mm Hg)	70 (65–74)	66 (62–70)	−4 (−8–1)	0.066	70 (66–74)	70 (66–74)	0 (−4–3)	0.830	−3 (−9–3)	0.214
MAP (mm Hg)	93 (89–97)	88 (84–93)	−4 (−9–1)	0.094	94 (90–98)	94 (89–98)	−1 (−5–4)	0.722	−3 (−9–4)	0.360
Temp. of patients (°C)	36.6 (36.5–36.6)	36.6 (36.6–36.7)	0.04 (−0.02–0.11)	0.189	36.6 (36.5–36.6)	36.5 (36.4–36.6)	−0.04 (−0.12–0.05)	0.369	0.08 (−0.04–0.20)	0.137
hs‐TnI (ng/l)	32.3 (22.1–43.3)	43.0 (27.9–58.1)	10.7 (0.8–20.6)	<0.001	45.6 (28.0–63.2)	43.2 (27.4–59.0)	−3.1 (−15.0–8.9)	0.250	9.8 (−2.6–22.1)	0.062
NT‐proBNP (pg/μL)	28.6 (13.6–43.5)	40.5 (22.8–58.3)	12.0 (1.5–22.4)	<0.001	46.4 (18.6–72.1)	50.7 (17.3–84.0)	4.3 (0.8–16.0)	<0.001	8.3 (−4.5–21.1)	0.322
Pulse	73 (69–77)	74 (69–78)	0.1 (−2.8–2.9)	0.573	72 (68–77)	73 (69–77)	0.0 (−3.3–3.3)	0.948	0.2 (−3.7–4.0)	0.674
PR interval in ECG	181 (165–196)	180 (169–193)	−1.3 (−15.7–13.0)	0.570	183 (170–197)	180 (168–192)	−3.1 (−10.0–3.7)	0.641	−6.1 (−22.5–10.4)	0.367
QRS interval in ECG	105 (97–114)	114 (104–123)	8.8 (3.6–14.0)	<0.001	107 (98–115)	114 (105–124)	7.3 (2.2–12.5)	<0.001	4.1 (−1.6–9.8)	0.316
QTC interval in ECG	443 (432–454)	451 (438–464)	9.8 (−2.3–21.8)	0.133	444 (434–454)	452 (440–464)	8.4 (−0.1–17.3)	0.061	7.0 (−6.6–20.6)	0.501
K^+^ (mEq/L)	5.1 (4.8–5.4)	3.4 (3.3–3.5)	−1.8 (−2.0–[−1.5])	<0.001	5.0 (4.7–5.4)	3.3 (3.2–3.4)	−1.7 (−2.1–[−1.3])	<0.001	0.01 (−0.35–0.36)	0.537
Ca^2+^ (mg/dl)	4.6 (4.5–4.7)	4.7 (4.6–4.8)	0.14 (0.03–0.25)	0.015	4.6 (4.4–4.7)	4.7 (4.6–4.8)	0.10 (−0.06–0.25)	0.228	0.03 (−0.16–0.22)	0.480

*Note*: Data are presented as the mean and 95% Confidence interval.

Abbreviations: BMI, body mass index; Ca, calcium; K, potassium; MAP, mean arterial pressure; Temp, temperature; ΔΔ, Δ routine dialysis ‐ Δ hypothermic dialysis.

^a^ Routine dialysis: before versus after.

^b^ Hypothermic dialysis: before versus after.

^c^ Δ routine dialysis versus Δ hypothermic dialysis.

d Weight is used as a proxy for ultrafiltration ‐uUltrafiltration volume = weight after the dialysis ‐ weight before the dialysis.

**TABLE 3:** Mean, 95% confidence interval of the differences (Δ) in mean arterial pressure (MAP) at four times of measurements according to type of dialysis

	Type of dialysis		
Time	Routine	Hypothermic	*p* ^a^
ΔMAP_1–2_	1.6 (−3.2–6.3)	2.6 (−1.1–6.3)	0.889
ΔMAP_2–3_	1.9 (−1.9–5.7)	1.1 (−2.5–4.8)	0.944
ΔMAP_3–4_	0.9 (−3.5–5.4)	−3.4 (−7.3–0.5)	0.123
*p* ^b^	0.973	0.287	

^a^ Routine dialysis versus hypothermic dialysis.

^b^ Repeated measures between three Δ in each type of dialysis.

## RESULTS

**FIGURE 1:**
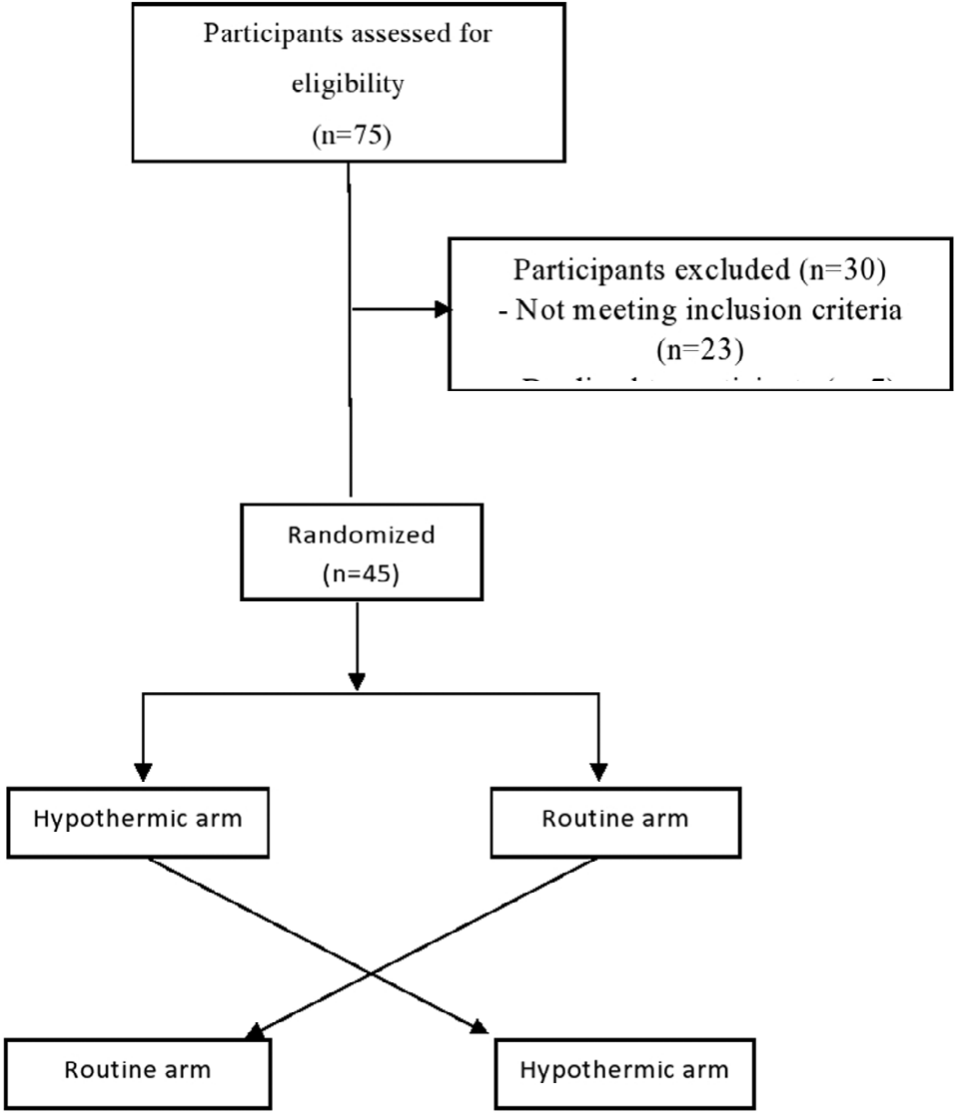
Study flow chart

Of 75 patients assessed for eligibility, 23 patients did not meet the inclusion criteria and 7 declined participating (Figure 1). Of the 45 patients recruited in the study, 60% were males and mean age was 64.5 years (Table 1). The median duration of dialysis duration was 2.2 years (range 0.3–10) (Table 1). Dialysis was performed three times per week on all patients. No adverse events (such as ischemic insults or myocardial events) requiring withdrawal from the study were reported. Two patients in the hypothermic arm and one patient in the routine arm reported feeling cold.

**FIGURE 2:**
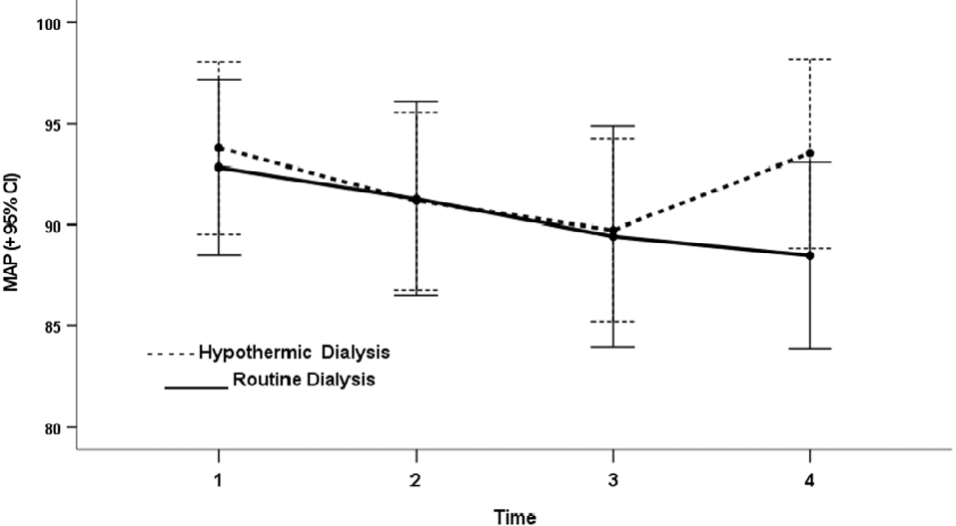
Mean arterial pressure (+95% confidence interval) at four times of measurements according to type of dialysis

During routine HD, systolic and diastolic blood pressures decreased by 5.0 ± 24.6 mmHg (*p* = 0.17) and 3.6 ± 12.7 mmHg (*p* = 0.08) respectively, compared with a decrease of 3.5 ± 27.3 mmHg (*p* = 0.6) and 0.4 ± 11.7 mmHg (*p* = 0.9) respectively in hypothermic dialysis, when comparing blood pressure measurements before with after HD (Table 2). Mean arterial blood pressure (MAP) decreased by 4 mmHg and by 1 mmHg in routine and hypothermic HD respectively, during the course of the dialysis (Figure 2). In addition, there were no statistically significant changes in MAP in different periods between the two arms (Table 3). The proportion of patients experiencing a decrease in systolic BP of more than 20 mmHg was similar in both arms (Table 4). No patients in either arm experienced a clinical event requiring nursing intervention. Neither arm experienced statistically significant changes in blood pressure (Table 2). Hs‐TnI levels at baseline were elevated in both groups (Table 2). However, hs‐TnI significantly increased during routine HD (+10.7 ng\ml, *p* < 0.01) but not in the hypothermic HD (−3.1 ng\ml, *p* = 0.25). NTproBNP significantly increased in the routine HD group (+12 pg\μl, *p* = 0.025) but not in the hypothermic HD group (+4.3 pg\μl, *p* = 0.4, Table 2). The difference in the delta between the two groups was however not significant for NTproBNP, and borderline for hs‐TnI (Table 2). No significant differences in hs‐TnI and Nt‐proBNP were observed in patients treated with antiplatelet drugs (such as aspirin, clopidogrel, ticagrelor, etc.) and in particular subgroups such as diabetic patients (*p* > 0.05). Significant differences were observed for the patients with CAD and CABG (*p* < 0.05) but not for the patients with previous PCI (*p* > 0.05).

Electrocardiograms (ECGs) were performed before and in the last 5 min of HD in both arms. Although QRS interval was significantly increased in both arms, no significant difference related to K^+^ and Ca^2+^ were observed when comparing the arms (Table 2).

**TABLE 4:** Percentages of IDH according to type of dialysis

	Type of dialysis	Routine	Hypothermic	
	Time	%	%	*p*
Sys ≤90	1	0	4.4	0.153
	2	4.4	6.7	0.645
	3	7.0	4.7	0.625
	4	10.3	2.4	0.141
	*p*	0.186	0.821	
ΔSys ≥20	ΔSys_1–2_	24.4	24.4	—
	ΔSys_1–3_	23.8	32.6	0.370
	ΔSys_1–4_	23.1	28.6	0.573
	*p*	0.989	0.701	
ΔMAP ≥10	ΔMAP_1–2_	24.4	15.6	0.292
	ΔMAP_1–3_	16.7	16.3	0.962
	ΔMAP_1–4_	20.5	26.2	0.547
	*p*	0.669	0.395	

## DISCUSSION

Our study results showed a tendency towards less rise in hsTnI and NTproBNP during hypothermic HD (35.5°C) as compared to routine HD (36.5°C). hsTnI raised significantly during routine dialysis but not hypothermic dialysis, and mean increase in NTproBNP was smaller in the hypothermic group. The difference between the two groups was however not statistically significant, although close to reaching significance for hsTnI. We noticed increase in hs‐TnI in the routine arm which is'nt seem to be related to hemoconcentration secondary to ultrafiltration since no changes in hs‐TnI were noted in hypothermic dialysis in comparison to routine dialysis and ultrafiltration (estimated by weight before and after dialysis) was similar for both kinds of dialysis.

Hs‐TnI is a sensitive biomarker for myocardial injury. Hs‐TnI levels are frequently elevated in the context of chronic kidney disease, which may reflect a repetitive cardiac injury during HD as it is associated with significant circulatory stress causing ischemic insults to multiple organs.^3^ As a result, there is no threshold hs‐TnI value that reliably identifies acute cardiac injury in HD patients. Troponins are more challenging to interpret for the diagnosis of acute myocardial infarction among CKD patients compared with the general population. The use of serial values to assess for a dynamic rise or fall in concentration to assess myocardial injury has been suggested.^10^ In one series of 670 consecutive dialysis patients who presented with chest pain or dyspnea, the area under the curve for receiver operating characteristic (ROC) based on the initial high‐sensitive cTnT was only 0.68 but improved to 0.9 with the addition of evaluation of relative change at 3 h and the optimal cut‐off value was 24%.^11^ Hypothermic dialysis seemed to induce a protective effect from injurious perfusion of the cerebral vascular beds, thus minimizing HD induced brain injuries,^12^ and significant reductions in both left ventricular mass and left ventricular end‐diastolic volumes.^1^ Mclntyre et.al showed preservation, or improvements, in important structural and functional cardiovascular abnormalities with dialysate cooling^12^ although the mechanism of action is unclear. It has been suggested that lowering body core temperature improves systemic vascular resistance and therefore improve hemodynamic stability.^8^ In line with existing evidence, our findings showed a tendency to less increases in cardiac injury markers using hypothermic HD compared with standard HD, although the absolute numbers were no different in both arms. Our study also suggests better hemodynamic stability in hypothermic HD as compared to routine HD with no difference in the frequency of intradialytic hypotension events but less intradialytic systolic blood pressure <90 mmHg. These differences were however not statistically significant. A recent systematic review demonstrated that fixed reduction of dialysate temperature decrease the incidence of intradialytic hypotension compared with routine dialysate.^13^

Natriuretic peptides are a well‐described family of hormones with a major role in sodium and body volume homeostasis. Several studies demonstrated that NT‐proBNP levels correlated better with LV function and hypertrophy than with volume status in HD.^14^ Furthermore, patients with high‐NT‐proBNP levels either predialysis or postdialysis had significantly higher total mortality.^15^, ^16^ Our patients had high levels of NT‐proBNP pre and postdialysis in both arms, but less changes were observed with hypothermic HD. Correlation between changes in NT‐proBNP levels during HD and cardiovascular mortality in HD patients is unclear.

The randomized, crossover design is a study strength. The dialysate composition for each patient was not available although most patients had a calcium dialysis solution of 1.25 mol/l with bicarbonate 34 mol/l, and the composition did not change from one cycle to the next. Since no other changes were made to the dialysis protocol and each patient were sequentially entered into each arm, the study essentially self‐controls for all factors except for dialysate temperature. Several potential limitations should however be noted. First, our study is single‐centre and the sample size relatively small. Second, compared with core temperature, measurement oral body temperature is less accurate and influenced by exposure to the external environment. Blood line temperature was not available. Third, blood pressure measurements were performed four times during each dialysis session. Additional measurements could have been more appropriate, specifically if we need to monitor for intradialytic hypotension events. Fourth, while troponin levels were checked at baseline and at the end of dialysis, the time elapsed may be insufficient to allow for troponin to rise significantly. Fifth, use weight change during HD as proxy to ultrafiltration and absence of actual ultrafiltration volume.

The decision not to lower dialysate temperature below 35.5° was based on clinical judgment and feedback from patients who reported a cold sensation and shivering. It perhaps elicits a greater response in terms of BP, TnI and NT‐proBNP but could potentially be problematic for the patients due to greater discomfort. There is no evidence suggesting either harm or benefits of lowering the dialysate temperature further. An individualized dialysate cooling approach based on patient response could be considered. Evidence of physiological and perceived responses from dialysis patients at different dialysate temperatures are needed.

In summary, our data suggest that compared with routine HD, hypothermic HD is associated with less changes in cardiac markers such as high‐sensitive troponin and NTproBNP. However, before cooled dialysate is considered for routine clinical practice, further studies are warranted to confirm our observation in larger cohorts, and to determine whether these changes actually lead to differences in clinical outcomes in terms of cardiac injury.

## FUNDING INFORMATION

The authors state that the study was done at their hospital without any external help. This research did not receive any specific grant from funding agencies in the public, commercial, or not‐for‐profit sectors.

## CONFLICT OF INTEREST

We do not have any conflict of interest.

## ETHICS STATEMENT

The study was approved by the Ethics Committee of Ziv Medical Center (0101‐17‐ZIV).
